# The Mummification of Pulp Tissue

**Published:** 1900-01-15

**Authors:** 


### DENTAL REGISTER.

Vol. LIV. JANUARY 15, 1900. No. 1.

### THE MUMMIFICATION OF PULP TISSUE.

Original and Reprint Articles, Editorially Assembled.

At this time there is probably no other subject undergoing as much experimental research as the practice of embalming portions of the pulp tissues, which can not be readily removed from intricate pulp canals, and we desire to present the subject to our readers in as complete and concise form as may be from the literature at command. It is not possible, without considerable time spent in examining early writings, to determine when and by whom the question was first agitated. Prof. Flagg says it was begun nearly half a century ago, when creosote was the embalming agent employed. Then oil of cloves was added, also sulphite of lime. He recommends a formula (see *Items of Interest*, page 930, Dec. 1899), which he has used satisfactorily for nearly twenty years:

℞. Oily (?) Carbolic Acid,
Oil of Cloves, aa ℥ss.

Mix, and add of the following sufficient to make a thick paste:

Sulphite of Lime,
Acetate of Morphine, aa. ʒj.

The more recent agitation of the subject, and in fact the beginning of the methods as at present practiced, seems to have been brought to notice through the efforts of Dr. Wilhelm Herbst, as set forth in a paper read before the New York State Dental Society, and published in the October, 1892, *Cosmos*, page 773, by Dr. C. F. W. Bodecker.

The method as here presented consisted of amputating the coronal portion of the pulp and burnishing into the cavity soft tin foil. The exposed pulp is first subjected to an application of cobalt, ninety-two parts, and cocain, eight parts, for two or three days, when it is removed with antiseptic precautions, and the coronal portion of the pulp amputated with a large, round, sterilized bur. The cavity is then cleansed of all debris with a bichloride wash and carefully dried with hot air. As large a cylinder of loosely-rolled tin foil as is admissible is then placed in the cavity and packed firmly into the cavity immediately covering the remaining pulp tissue and root canals. The intent being to hermetically seal in the pulp by the close adaptation of the tin. It is best packed by a large, smooth, round engine burnisher. Then over this tin capping or seal any kind of filling required may be placed to complete the filling of the cavity. To avoid discoloration by the tin, gold may be used in teeth exposed when the mouth is open.

The theory on which this practice is based is, or seems to be, that the vascular portion of the pulp being largely removed by the operation, and the remainder left in a sterile condition by careful antiseptic manipulation remains in the roots not liable to attack from decomposing influences. It is claimed that in many cases so treated the pulps are not entirely poisoned by the cobalt, and that they revive and keep up the nutrition of the tooth tissues for long periods after the operation. Teeth so treated have been extracted and examined microscopically afterward, showing signs of vital function after the treatment. This point has been much questioned, and the practice of this method has never had a large following. But there is really no valid objection to this practice, as the results outlined would, in many cases, justify the practice if all the steps are antiseptically taken. The more rational theory seems to be, however, that the arsenic in the cobalt, and the bichloride of mercury, completely kill all remains of the pulp and sterilize it so that conditions favorable to its fermentation are not present, and the tooth remains comfortable for a sufficiently long period to justify the procedure.

Many able writers and conservative practitioners have condemned the practice because it is not in accord with accepted principles of practice. (See article by Dr. Otto Arnold, *Cosmos*, 1893, page 126, and by Dr. A. W. Harlan, *Cosmos*, 1893, page 169, and the discussion of the same on page 221.) Dr. Wm. E. Christensen, in *Cosmos*, 1893, page 363, commends the practice, as advocated by Herbst, and justifies his position by a considerable practice.

### EMBALMING BY MEDICATION.

The first systematic effort in this direction was probably made by Dr. Adolph Witzel, of Essen, Germany, and the following extract from a paper contributed to the Columbian Dental Congress will not only give us its history, but also the ideas of Prof. W. D. Miller, its author.

Witzel's method was to apply arsenious acid to inflamed pulps for twenty-four hours and then amputate the coronal portion and fill the cavity thus made with a paste of carbolic acid and oxide of zinc, and complete the operation with amalgam or cement. Witzel supposed the stump healed up, or shrank to antiseptic or sterilized threads under the action of the paste. He also used other antiseptics, bichloride, etc.

Witzel made his first report in 1874, and in 1886 he reported having made several thousand operations up to that time, with only 3 percent of failures.

In 1888 Baume suggested a method of "pickling" pulp stumps with a solution of alum and borax, and reported two hundred cases without a single failure. The permanent results were, however, not satisfactory, as the weak character of the antiseptics used was not sufficient to maintain sterile conditions for very long periods, and this method was soon abandoned.

The Herbst method, although more of an operative procedure, was probably the outgrowth of this method, or the one advocated by Witzel.

Dr. Miller believes that the success of each of these methods depends largely on the character of the antiseptics used, especially with reference to their chemical reactions. The essential qualities he enumerates as follows:

1st. Antiseptics of great power should be used.

2d. They should be soluble and diffusible to insure impregnation of the entire pulp.

3d. Excessively soluble substances should not be used, as their action would be transient.

4th. A coagulant may be used, although not essential; a coagulant is much preferable to a digestant or saponifying agent.

5th. Non-irritants should be used rather than irritants, especially such as would penetrate the dentin or apical foramen.

6th. It should not discolor the teeth.

7th. Solids or semi-solids are preferable to liquids.

Dr. Miller refers to the difficulties of finding a remedy which will measure up to all these requirements, and refers to an article which will be helpful in this search, printed in the *Dental Cosmos* for August, 1890, page 589.

In this article, after a research of five hundred experiments, he concludes by dividing dental antiseptics into the three groups as follows:

1st. Those which possess in a high degree the power of imparting antiseptic qualities to root pulps. They are: cyanide of mercury, bichloride of mercury, diaphtherin, sulphate of copper, salicylate of mercury, oil of cinnamon, ortho-kresol, carbolic acid, trichlorphenol, chloride of zinc. The last four are inferior to the others, they penetrate the pulp rapidly, but lack antiseptic power, and are so diffusible as to disappear from the pulp in a few weeks.

2d. Those of doubtful value, such as thymol, salicylic acid, eugenol, campho-phenique, hydronaphthol, alpha and beta naphthol, acetico tartrate of aluminum, some essential oils, resorcin, thallin, sulpho-carbolate of zinc, oil of birch, iodide of sodium, nitrate of sodium, etc.

3d. Those nearly or quite worthless, such as iodoform, basic anilin coloring matters, borax, boracic acid, dermatol, europhen, chloride of lime, peroxide of hydrogen, sozoiodol salts, iodol, tincture of iodine, camphor spirits, napthaline, etc.

Dr. Miller experimented on the basis of this research in between four hundred and five hundred cases, using a small tablet of the following composition:

No. 1.

R. Bichloride of Mercury, grain $\frac{1}{6}$,

Boracic Acid, " $\frac{1}{3}$.

or

R. Bichloride of Mercury, grain $\frac{1}{6}$,

Chloride of Sodium, " $\frac{1}{3}$.

The method of using is to devitalize the pulp with arsenic or other suitable preparation, then thoroughly cleanse the pulp chamber and apply one of the above tablets and crush it to a powder with a plugger or other suitable instrument and moisten with sterilized water, allow it to penetrate the canals and saturate the pulp remains. A layer of tin or gold foil is then placed in the cavity over the canals and the filling, cement or amalgam inserted directly over it. In 30 percent of the cases so treated severe pain, sometimes soreness and swelling, resulted from the application, but subsided in a day or two; in no case did suppuration follow. The irritation was doubtless due to the action of the bichloride on the peridental structures. To overcome the penetration of the bichloride the formula was changed to

No. 2.

R. Bichloride of Mercury,
Thymol, aa. grs. $\frac{1}{10}$.

This tablet is used in the same way, the thymol being used to prevent the excessive diffusion of the bichloride and also to increase its sterilizing power and to increase the permanence of the application. The use of this tablet seems not to produce the irritable results of No. 1.

No. 3. Another formula used was:

R. Bichloride of Mercury,
Thymol,
Tannin, aa grains $\frac{1}{12}$.

The tannin is designed to harden or cure the tissue and also to prevent excessive penetration. It however discolors the tooth to some extent.

Dr. Miller also commends the cyanide of mercury and thymol in $\frac{1}{12}$ grain quantities, also the salicylate of mercury, and had good results from diaphtherin, applied in pure form alone. Of the essential oils, cinnamon takes the first place.

In the *Dental Cosmos* for November, 1895, Dr. Soderberg, of Sydney, Australia, makes a valuable contribution to this subject, particularly from the practical side, and we make the following liberal extract from his report:

